# Venoplant Effect in the Management of the Post-operative Oedema in Plastic Surgery: Results of a Randomized and Controlled Clinical Trial

**DOI:** 10.1007/s00266-018-1108-z

**Published:** 2018-03-05

**Authors:** Francesco D’Andrea, Luca D’Andrea, Ercole Manzi

**Affiliations:** Unit of Plastic, Reconstructive and Aesthetic Surgery, University “Federico II”, Naples, Via S. Pansini 5, 80131 Naples, Italy

**Keywords:** Oedema management, Swelling management, Anti-oedema drugs, Aesthetic surgery

## Abstract

**Background:**

Post-operative oedema and ecchymosis represent early post-operative complications, impacting negatively on the final aesthetic outcome of each surgical procedure. In particular, such complications are very frustrating for patients and—sometimes—are difficult to be managed by surgeons. Several strategies are available for managing oedema, although some side effects have been reported. A new promising compound for the management of oedema is Venoplant, and this study aims to assess its effectiveness in decreasing post-operative oedema.

**Methods:**

Patients were randomly allocated for receiving three different treatments: (1) Venoplant tablets and Venoplant gel; (2) only Venoplant tablets; and (3) not treated with Venoplant. The aesthetical outcome has been evaluated using the Global Aesthetic Improvement Scale (GAIS), compiled by both patient and clinician. The GAIS scale was administered several times: the day following the surgical procedure (*T*0) after 3 days (*T*1), after 7 days (*T*2), after 15 days (*T*3) and after 1 month (*T*4).

**Results:**

Forty-three patients participated in the study. According to patient’s evaluations, at *T*0 in Group 1 and in Group 2 a significant statistical difference was found compared to the control group (*p* < 0.001 and *p* < 0.05, respectively). Over time, a significant reduction in swelling and ecchymosis was reported by patients treated with Venoplant (tablets alone or in combination with gel) compared to the control group. According to the physician’s assessment, during the different time points of evaluation, a significant reduction in post-operative oedema in Group 1 and in Group 2 compared to the control group was found.

**Conclusion:**

Venoplant represents a valid therapeutic strategy for the management of post-operative oedema, guaranteeing a good level of patient satisfaction, in the absence of common side effects which are often associated with other therapies.

**Level of Evidence I:**

This journal requires that authors assign a level of evidence to each article. For a full description of these Evidence-Based Medicine ratings, please refer to the Table of Contents or the online Instructions to Authors www.springer.com/00266.

## Introduction

Post-operative oedema and ecchymosis represent early post-operative complications, impacting negatively on the final aesthetic outcome of each surgical procedure. In particular, these complications are associated with delayed post-surgical recovery and achievement of the definitive aesthetic result, with high levels of dissatisfaction reported by patients [1]. Among these complications, oedema represents the most annoying and disabling effect, delaying the patient’s return to routine activities.

Different methods are used to decrease post-operative oedema from the use of drain [2–4], compression garments [5] and ice bags [6] to medical therapies based on corticosteroids and non-steroidal anti-inflammatory drugs (NSAIDs) [7].

Another additional therapeutic option is based on anti-oedema drugs, whose role in aesthetic plastic surgery is rather controversial.

This study shows the use of Venoplant tablets and Venoplant gel (Aesculapius, Brescia, IT).

Venoplant tablets are food supplements with three active ingredients that synergize: Coumarin (32 mg), *Centella asiatica* (15 mg) and micronized diosmin (300 mg).

The coumarin blocks the synthesis of pro-inflammatory prostaglandins and increases the proteolytic activity of the macrophages to decrease interstitial protein accumulation, one of the main causes of oedema [8].

*Centella asiatica* stimulates the tropism of the connective tissue, by increasing collagen synthesis and decreases capillary permeability [9, 10].

The diosmin decreases capillary permeability, increases venous tone and reduces tissue fluid accumulation [11, 12].

Venoplant gel is a formulation for topical use containing: escin (1 g), heparin (4000 U.I), coumarin (0.1 g) and *Centella* (0.1 g).

The escin decreases capillary permeability with an antiedemigenic, anti-inflammatory and venotonic action [13].

The heparin is a mucopolysaccharide able to keep water in interstitial spaces, accelerates the removal of leftover material from metabolism and inflammatory processes; moreover, it helps absorption of active ingredients [14].

This study aims to assess the effectiveness of Venoplant in decreasing post-operative oedema and ecchymosis in patients undergoing aesthetic plastic surgery (i.e., additive mastoplasty, blepharoplasty, rhinoplasty and facelift). In particular, we aim to demonstrate that the association between the oral and topical formulation of Venoplant has a synergic effect, allowing a faster reduction in the oedema and the ecchymosis.

## Materials and Methods

From September 2015 to December 2017, all patients aged between 18–65 years old, able to express their informed consent referred for an intervention of aesthetic plastic surgery (additive mastoplasty, blepharoplasty, rhinoplasty, facelift) were recruited. Exclusion criteria from the study were: 1) patients suffering from disorders of coagulation,  treated with antiplatelet and/or anticoagulant oral medications; patients who have suspended treatments with antiplatelet and/or anticoagulant oral drugs for less than one month. According to a randomized procedure, patients were allocated into three groups: 1. patients receiving Venoplant tablets and Venoplant gel; 2. patients receiving only Venoplant tablets; 3. patients not treated with Venoplant (considered as the control group). Assessment procedures were implemented by a blinded researcher, who did not join the randomization. Patients allocated in Group 1 and Group 2 received 2 tablets per day, from two days prior to the surgery until eight days after the surgery. Patients allocated in Group 1 started the treatment with Venoplant gel from the day following the surgical procedure 2 times/day, until the ecchymosis was resolved. All patients had to wear compression garments at the same time (if allowed according to the type of surgical procedure), and to use ice bags during the 3 hours following the surgical operation.

The aesthetical outcome of the treatments was evaluated using the Global Aesthetic Improvement Scale (GAIS) (Table 1), compiled by both patient and clinician.

**Table 1:** GAIS

Score	Evaluation	Description
3	Considerable improvement	Excellent aesthetic result
2	Significant improvement	Significant aesthetic improvement compared to the initial condition but not the best one for the patient
1	Small improvement	Clear aesthetic improvement compared to the initial condition
0	Any improvement	The condition is unchanged compared to the initial one
− 1	Small deterioration	The condition has slightly worsened compared to the initial one
− 2	Significant deterioration	Significant aesthetic deterioration compared to the initial condition
− 3	Considerably worsened	Considerable deterioration compared to the initial condition

The main outcome of the study was the Global Aesthetic Improvement Scale (GAIS) score. The GAIS score ranges from − 3 (clinical condition considerably worsened) to + 3 (clinical condition considerably improved) (Table 1). The GAIS scale was administered to patients and to the clinician several times: the day following the surgical procedure (*T*0), after 3 days (*T*1), after 7 days (*T*2), after 15 days (*T*3) and after 1 month (*T*4). The initial condition refers to the day of the surgical procedure.

### Statistical Analysis

Descriptive statistics, such as frequency counts, means and standard deviation were calculated as appropriate. To assess differences among the three groups for the GAIS score, ANOVA analysis with Bonferroni correction was implemented. Moreover, to evaluate the change in GAIS score over time, a repeated measure analysis was implemented.

All the statistical analyses were performed using the SPSS software, version 24.0. The level of statistical significance was set at *p* < 0.05.

## Results

Fifty-eight patients were invited to take part to the experimental study. Fifteen patients refused to participate with a final sample of 43 patients. They were mainly female (83.7%, *n* = 36), aged between 18 and 65 years old (M: 36.2; s.d.: 14.3). As regards the surgical procedures received by patients, the majority underwent an additive mastoplasty procedure (34.9%, *n* = 15); 16.3% (*n* = 7) received a rhinoplasty procedure; 14% (*n* = 6) underwent a mastopexy procedure with breast implants; and 11.6% (*n* = 5) of patients were treated with a mini-facial lift. In the remaining cases, five procedures of replacement of breast implants (11.6%) and five blepharoplasty (11.6%) procedures were implemented.

The recruited patients were randomly allocated to the three groups. In particular, sixteen patients were allocated to Group 1 (patients receiving Venoplant tablets and Venoplant gel); fourteen patients to Group 2 (patients receiving only Venoplant tablets); while the remaining thirteen patients received treatment as usual. Patients from Groups 1 and 2 reported full compliance with the prescribed treatment (according to time scheduling and dosage, as previously described).

According to patient’s evaluations, at *T*0 between Group 1 (patients treated with Venoplant tablets and Venoplant gel) and Group 3 (Control group) a significant statistical difference was found (*p* < 0.001) (− 1.3 ± 0.6 vs − 2.2 ± 0.7), as well as between Group 2 and Group 3 (*p* < 0.05) (− 1.5 ± 0.5 vs − 2.2 ± 0.7). At *T*1 (three days after the surgery), significant statistical differences were found between Group 1 and Group 3 (*p* < 0.01) (− 1.6 ± 0.6 vs − 2.5 ± 0.7) and between Group 2 and Group 3 (*p* < 0.01) (− 1.6 ± 0.7 vs − 2.5 ± 0.7). After one week from surgery (*T*2), patients from Group 1 (Venoplant tablets and gel cream) and from Group 2 (only Venoplant tablets) reported a statistically significant reduction in oedema and ecchymosis compared to the control group (Group 1: − 0.1 ± 0.9; Group 2: 0 ± 0.7; Group 3:− 1.2 ± 0.6; *p* < 0.0001). This improvement was also found two weeks after the surgical procedure (*T*3) (*p* < 0.0001), and 1 month later (*T*4) (*p* < 0.001 for Group 1 vs Group 3 and *p* < 0.0001 for Group 2 vs Group 3).

According to the clinician’s assessment, at *T*0 statistically significant differences were found between Group 1 and Group 3 (*p* < 0.05) (− 1.1 ± 0.7 vs − 1.8 ± 0.7). In each of the subsequent time points of evaluation, a significant reduction in post-operative oedema in Group 1 and in Group 2 compared to the control group was found (Table 2).

**Table 2:** ANOVA with Bonferroni corrections

	*T*0	*T*1	*T*2	*T*3	*T*4
	M (ds)	M (ds)	M (ds)	M (ds)	M (ds)
Group 1					
Gais P	− 1.3 (0.6)^a^	− 1.6 (0.6)^c^	− 0.1 (0.9)^e^	1.6 (0.8)^e^	2.8 (0.6)^a^
Gais M	− 1.1 (0.7)^g^	− 1.4 (0.5)^e^	0.3 (0.8)^g^	1.7 (0.7)^a^	2.8 (0.4)^e^
Group 2					
Gais P	− 1.5 (0.5)^b^	− 1.6 (0.7)^d^	0.0 (0.7)^f^	2.1 (0.7)^f^	2.9 (0.4)^f^
Gais M	− 1.4 (0.6)	− 1.7 (0.5)^b^	0.3 (0.8)^b^	2.1 (0.8)^f^	2.9 (0.4)^f^
Group 3					
Gais P	− 2.2 (0.7)^a,b^	− 2.5 (0.7)^c,d^	− 1.2 (0.6)^e,f^	0.2 (0.7)^e,f^	2.0 (0.6)^a,f^
Gais M	− 1.8 (0.7)^g^	− 2.2 (0.6)^e,b^	− 0.6 (0.8)^g,b^	0.5 (0.8)^a,f^	1.9 (0.6)^e,f^

^a^ANOVA with Bonferroni correction, Group 1 versus Group 3, *p* < 0.001

^b^ANOVA with Bonferroni correction, Group 2 versus Group 3, *p* < 0.05

^c^ANOVA with Bonferroni correction, Group 1 versus Group 3, *p* < 0.01

^d^ANOVA with Bonferroni correction, Group 2 versus Group 3, *p* < 0.01

^e^ANOVA with Bonferroni correction, Group 1 versus Group 3, *p* < 0.0001

^f^ANOVA with Bonferroni correction, Group 2 versus Group 3, *p* < 0.0001

^g^ANOVA with Bonferroni correction, Group 1 versus Group 3, *p* < 0.05

**Fig. 1:**
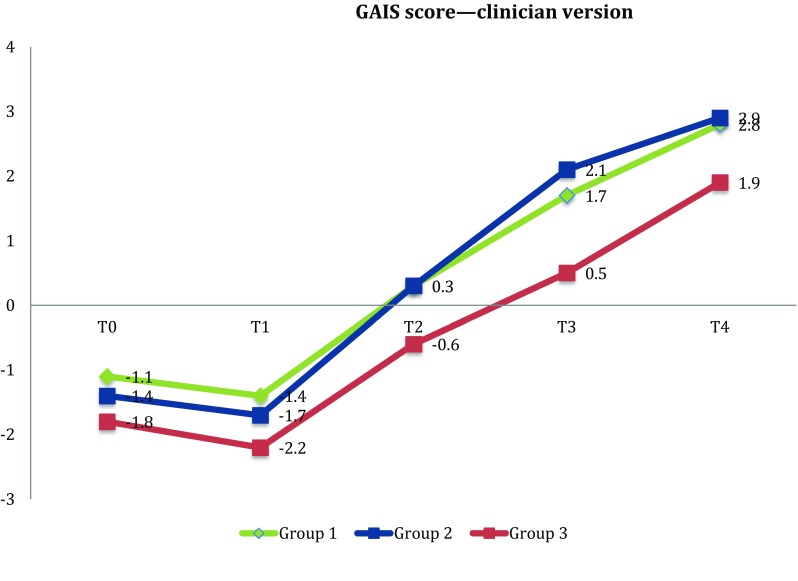
Repeated measures analysis—Effects of different treatments on GAIS score—clinician version (*p* < 0.05)

**Fig. 2:**
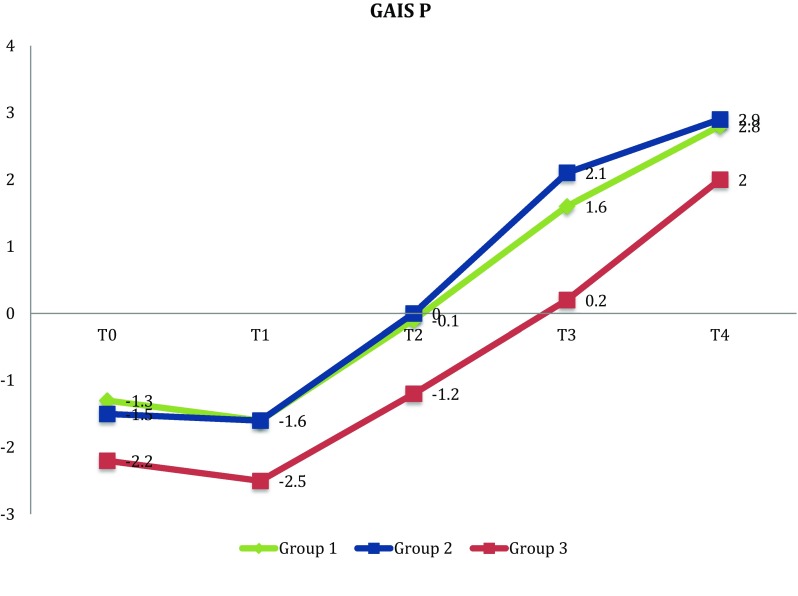
Repeated measures analysis—Effects of different treatments on GAIS score—patient version (*p* < 0.05)

According to the repeated measures analysis, a significant reduction in oedema and ecchymosis over time was found (*p* < 0.0001), both according to patient and clinician evaluations. A significant interaction between time point and the considered treatment was found, in particular patients of Group 1 and Group 2 showed a significant reduction in oedema compared to those of Group 3 (Figs. 1, 2).

Moreover, the type of surgical procedure did not show any significant impact on the effect of the experimental treatments.

## Discussion

**Fig. 3:**
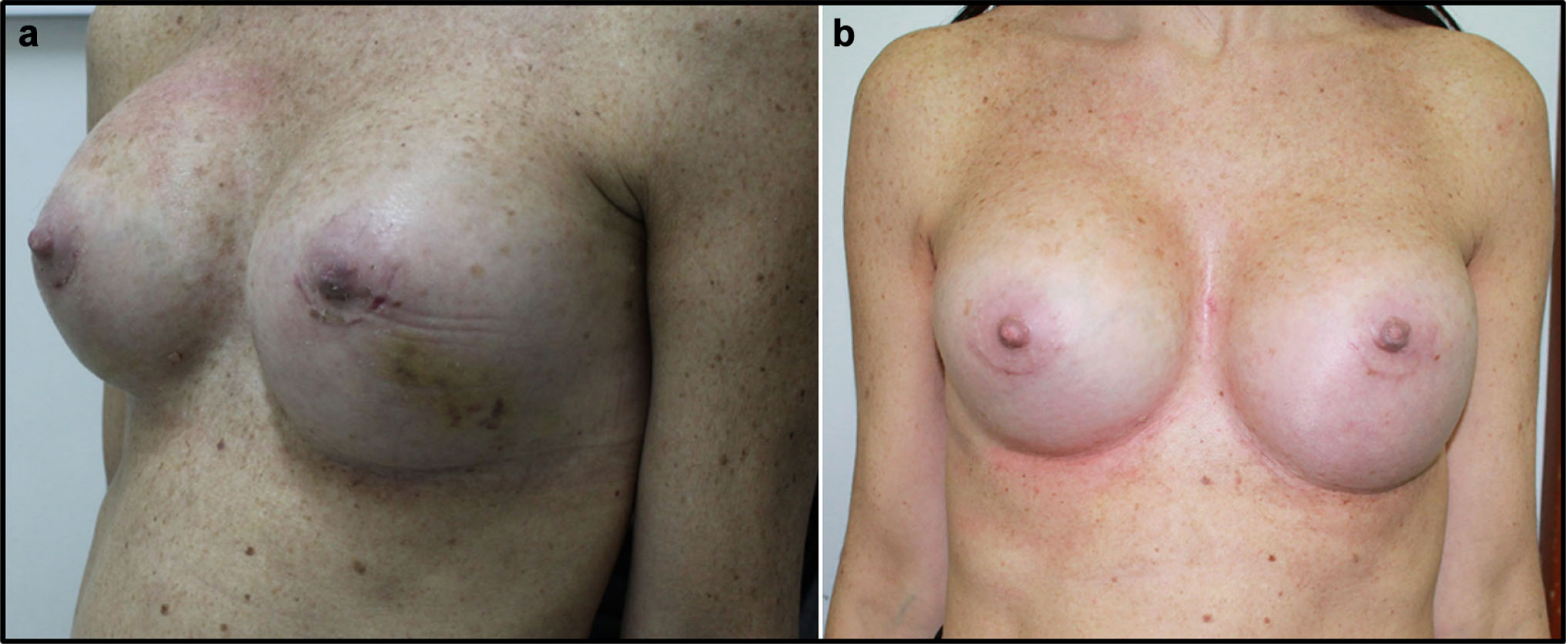
**a** Patient from Group 1, seven days after additive mastoplasty. **b** Patient from Group 1, 15 days after additive mastoplasty

**Fig. 4:**
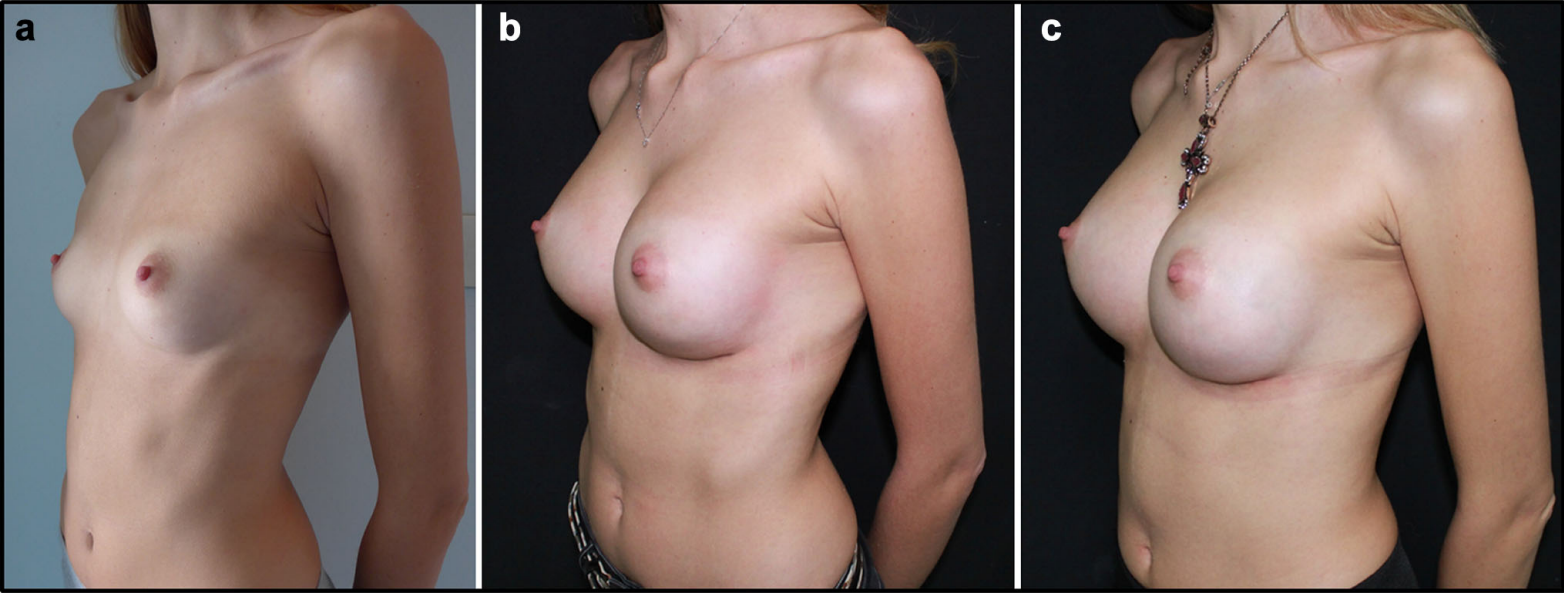
**a** Patient from Group 1, before additive mastoplasty. **b** Patient from Group 1, 15 days after additive mastoplasty. **c** Patient from Group 1, one month after additive mastoplasty

**Fig. 5:**
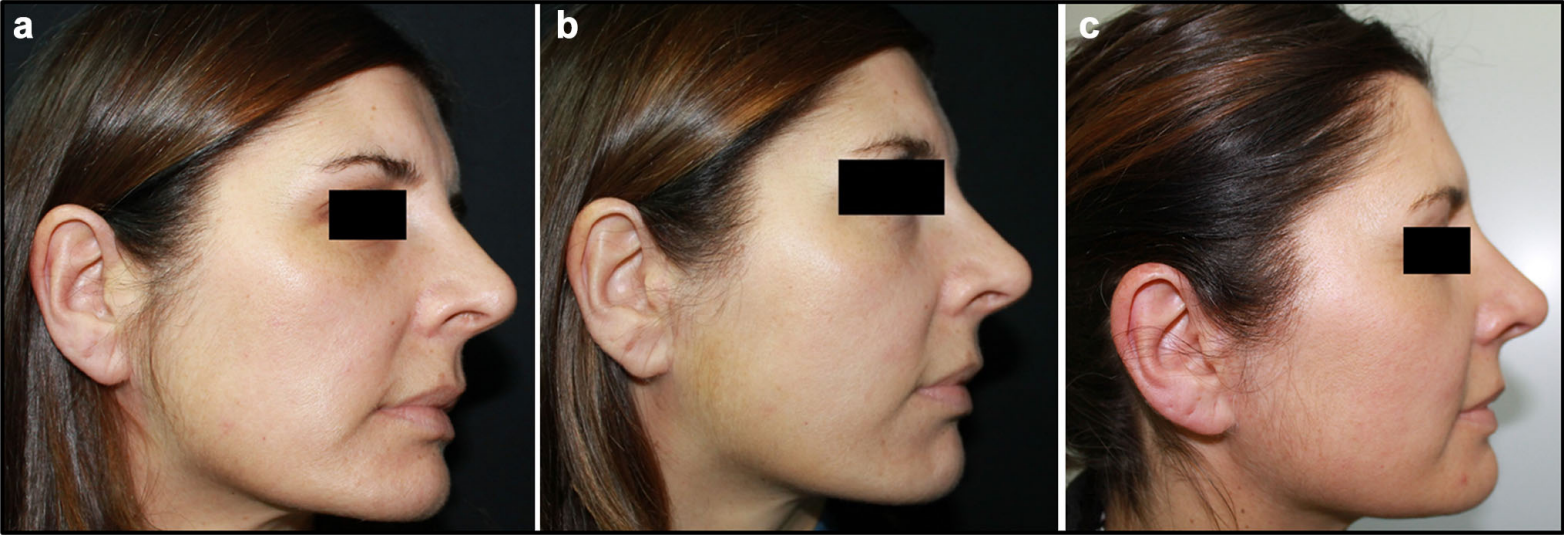
**a** Patient from Group 1, before III mid-facelift procedure. **b** Patient from Group 1, 15 days after III mid-facelift procedure. **c** Patient from Group 1, one month after III mid-facelift procedure

**Fig. 6:**
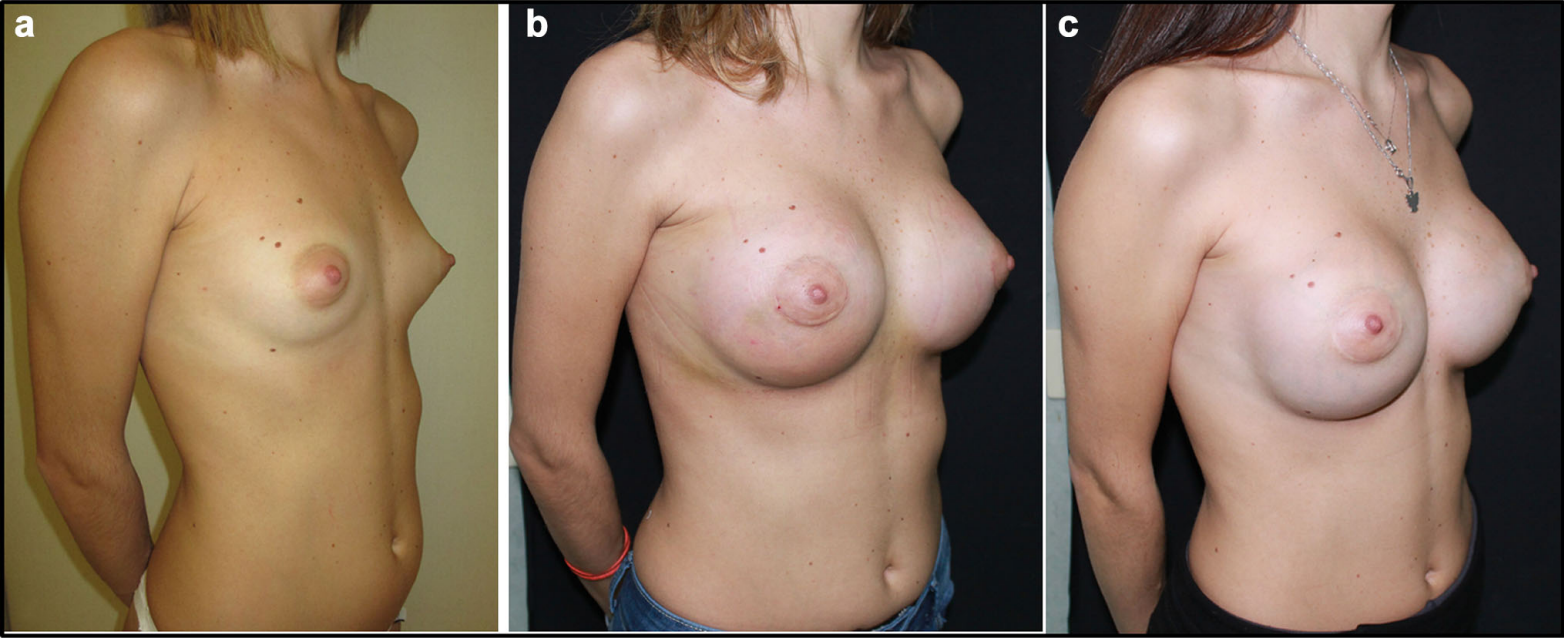
**a** Patient from Group 2, before additive mastoplasty. **b** Patient from Group 2, 15 days after additive mastoplasty. **c** Patient from Group 2, one month after additive mastoplasty

**Fig. 7:**
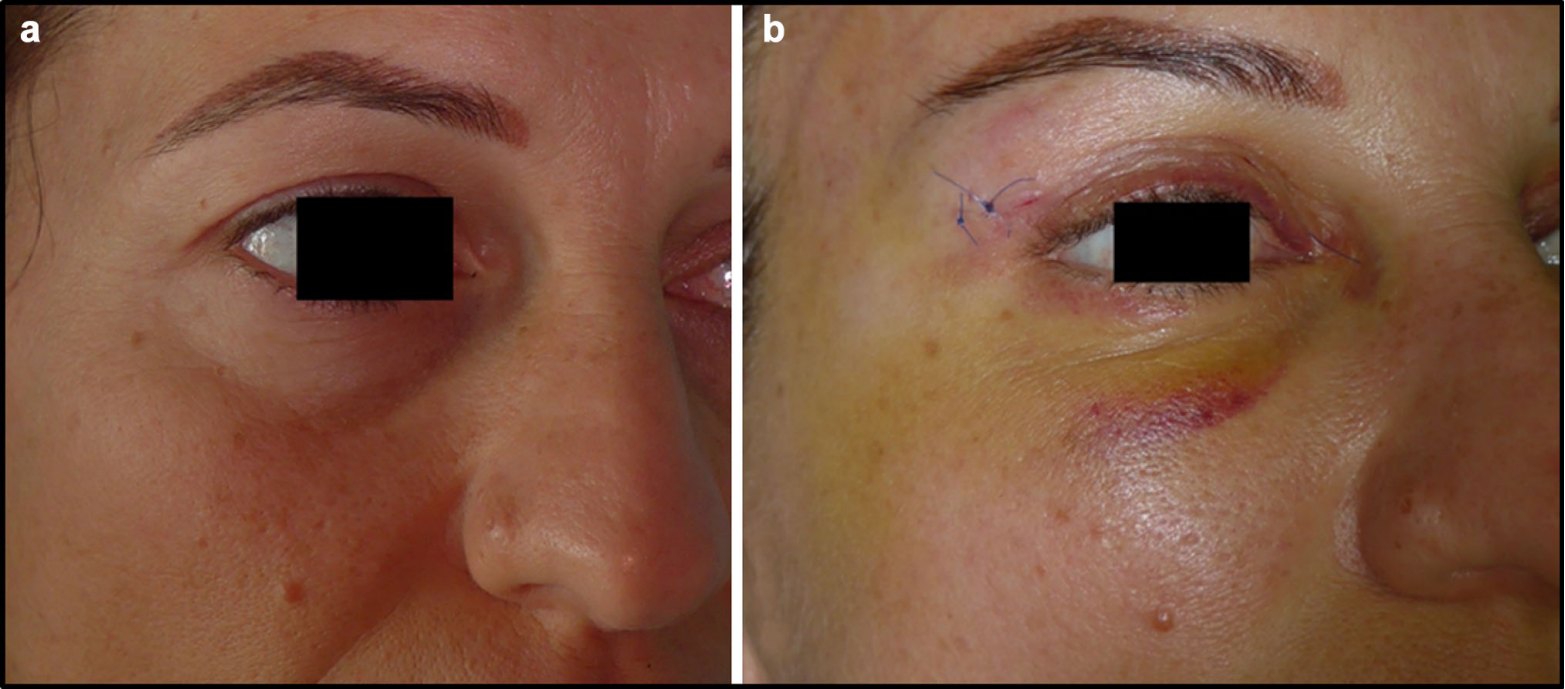
**a** Patient from Group 2 before blepharoplasty. **b** Patient from Group 2, three days after blepharoplasty

**Fig. 8:**
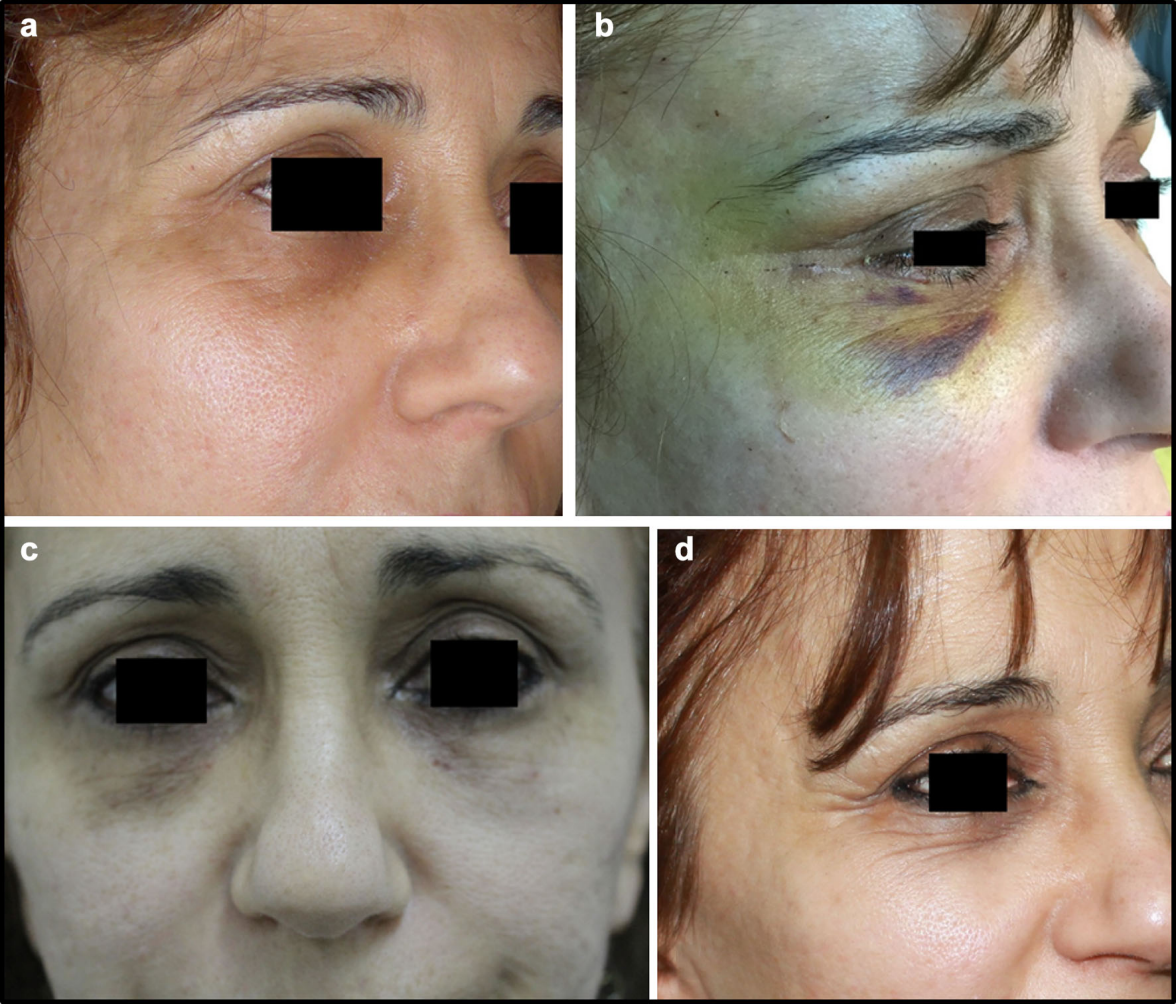
**a** Patient from the Control Group, before blepharoplasty. **b** Patient from the Control Group, 3 days after blepharoplasty. **c** Patient from the Control Group, 15 days after blepharoplasty. **d** Patient from the Control Group, one month after blepharoplasty

**Fig. 9:**
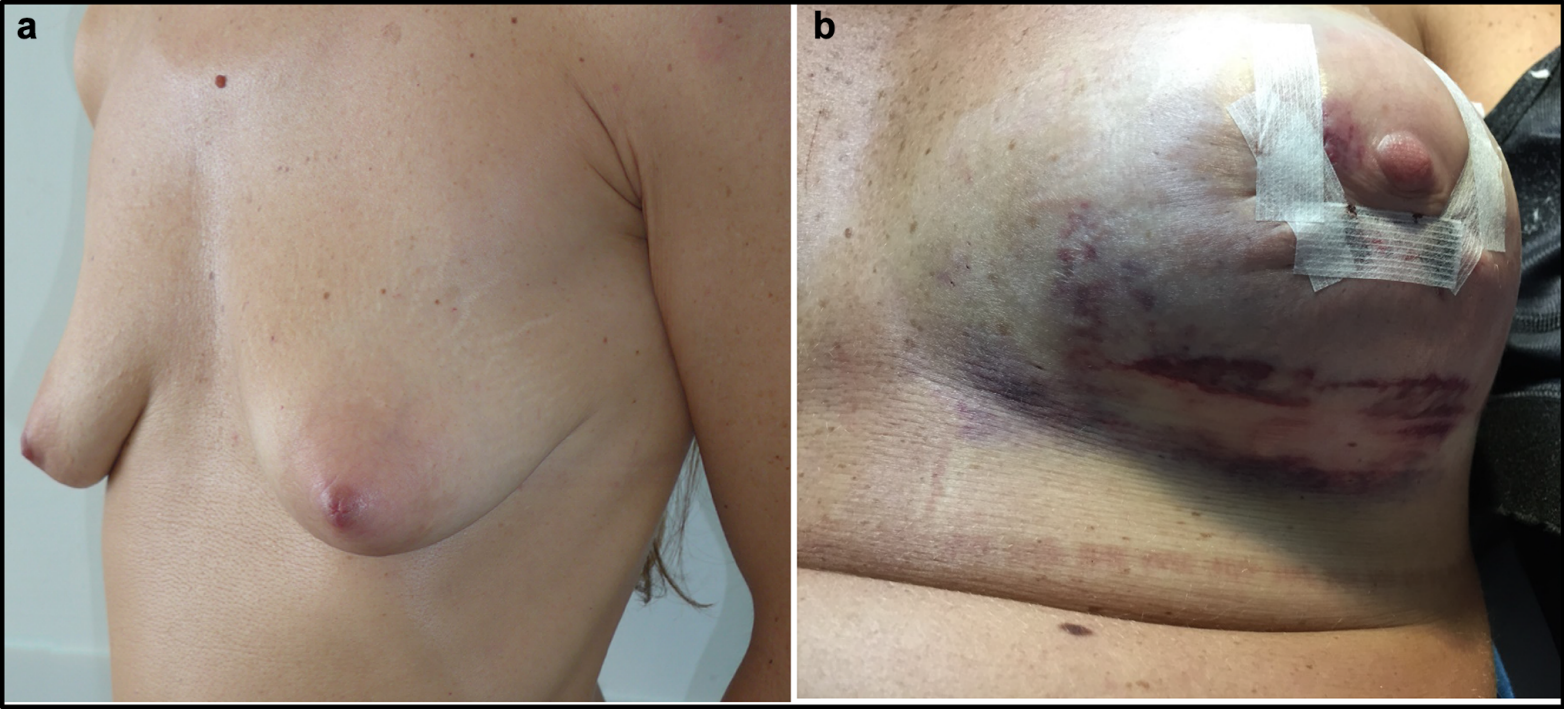
**a** Patient from the Control Group before additive mastoplasty. **b** Patient from the Control Group, seven days after the additive mastoplasty

Management of post-operative oedema is a significant problem after any kind of surgical procedure. This complication has a central role in aesthetic surgical procedures, because patients want to return to their daily routine soon after the intervention. Moreover, post-operative oedema reduction allows a faster achievement of the final aesthetic result. This study represents the first in the field of aesthetic surgery aiming to manage post-operative oedema using food supplements (Figs. 3, 4, 5, 6, 7, 8, 9). In previous studies in orthognathic surgery, Tozzi at al. [15] highlighted that using food supplements with antiedemigenic properties (such as Venoplants) guarantees a reduction in the prescription of corticosteroids.

In our study, even by using compressive garments (if allowed by the surgical procedure) and ice bags, corticosteroids were not prescribed, highlighting the positive effect of using Venoplant. Many clinical trials have been conducted to demonstrate corticosteroid effectiveness in treating post-operative oedema in aesthetic surgery. Coroneos et al. [16] demonstrated that a perioperative single dose of corticosteroids is able to decrease post-rhinoplasty oedema one day after the operation without increasing bleeding; but this improvement is not visible one week after the operation. Other studies reported contrasting results: Hatef et al. [17] found oedema reduction after 7 days, Youssef et al. [18] showed that there are no visible improvements 3 days after the operation, whereas Pulikottil et al. [19] concluded that oedema and ecchymosis reductions are transitory, and the risk of complications linked to the use of corticosteroids is higher than the benefits. In particular, in the case of rhinoplasty surgical procedures, the risks of side effects (the possible suppression of the hypothalamic-pituitary, avascular necrosis, wound infections, diabetes, hyperlipidaemia, peptic ulcers and allergic reaction) of a corticosteroid-based treatment reduce the possibility of adopting such a strategy.

The adoption of corticosteroids in facial aesthetic surgery showed no significant effectiveness in preventing oedema and ecchymosis after the surgical operation. Regarding the use in breast aesthetic surgery, Romundstad et al. [20] demonstrated the effectiveness of a perioperative single dose of corticosteroids in decreasing pain, emesis and post-operative fatigue but they did not provide any data regarding the management of oedema. Consequently, corticosteroid use appears to be unjustified.

The use of compressive garments in breast aesthetic surgery is a constant practice in decreasing post-surgical oedema and haematoma risk, but Nathan and Singh [21] recently found that compression has a limited effect in the management of post-surgical oedema in breast surgery and very often these garments cause patient discomfort.

Ice bag application is widely used to decrease post-surgical oedema [22, 23], but especially in the case of facial surgery, they can frequently cause headaches. The use of drainage is also controversial, in fact, according to Jones et al. [24] during facial-lifting procedures the use of drainage reduces ecchymosis with no significant advantage for controlling oedema. According to Araco et al. [25], the use of drainage during additive mastoplasty surgical procedures reduces post-operative oedema, but it increases the risk of infection of breast implants significantly.

In our study, the use of Venoplant showed no complications or side effects, guaranteeing great compliance by the patient.

Although no statistically significant differences among those receiving only Venoplant tablets and those treated with Venoplant tablets and Venoplant gel were found, the high level of satisfaction reported by patients should be highlighted. In fact, patients received both Venoplant formulations reported a faster reduction in ecchymosis. From a longitudinal perspective, a progressive reduction in oedema was found both in physician evaluations and in patient evaluations. Such a finding is not always guaranteed by other therapies used in the management of post-operative oedema and it could have contributed to good patient compliance, and consequently good management of oedema and ecchymosis.

This study has some limitations which need to be acknowledged. In particular, the small sample size, the heterogeneity in surgical procedures and the adoption of only a self-reported assessment tool (without objective measurements) could have biased the generalizability of our results. In particular, it could be useful to implement studies with a larger sample size for confirming these preliminary findings, also using radiological techniques to define the recovery process in a more precise manner. The adopted statistical methodology—with the development of an analysis for repeated measures—and the study design—with the presence of a control group—guarantee good reliability of our results, although it will be necessary to promote further studies with larger sample sizes and a longer follow-up periods.

In conclusion, Venoplant—a food supplement with antiedemigenic properties—represents a valid therapeutic strategy for post-operative oedema management, by guaranteeing a good level of satisfaction for patients and clinicians, in the absence of common side effects which are often associated with other available therapies.
